# Postoperative ileus in colorectal surgery: is there any difference between laparoscopic and open surgery?

**DOI:** 10.1093/gastro/got008

**Published:** 2013-03-28

**Authors:** Mehdi Fesharakizadeh, Diana Taheri, Shahaboddin Dolatkhah, Steven D. Wexner

**Affiliations:** ^1^Faculty of Medicine, Islamic Azad University, Najaf Abad Branch, Isfahan, Iran, ^2^Department of Colorectal Surgery, Cleveland Clinic Florida, Weston, Florida, USA, ^3^Department of Pathology, Faculty of Medicine, Isfahan University of Medical Sciences, Isfahan, Iran and ^4^Medical student, Faculty of Medicine, Isfahan University of Medical Sciences, Isfahan, Iran

**Keywords:** outcomes, postoperative complication, colorectal surgery, laparoscopic surgery, open surgery, ileus

## Abstract

**Background:** Postoperative ileus is a major complication of patients undergoing abdominal surgery. The purpose of this study was to determine the effects of operative time and the method of surgery on postoperative ileus.

**Methods:** After institutional review board approval, 121 patients were studied in two groups. Group 1 consisted of 86 patients with colorectal cancers and Group 2 included 35 patients with diverticulitis. Various surgical procedures were performed in both groups. In all patients, the nasogastric (NG) tube was removed after termination of surgery. Clear liquids were offered commencing on the first postoperative day, followed by a regular diet as tolerated. GI-1 was the postoperative time to toleration of clear liquids, whereas GI-2 was the postoperative time to first bowel movement or flatus and toleration of a regular diet. Statistical analysis was performed using a linear regression model by disease with the first bowel movement or flatus as the dependent variable and operative time and category as explanatory variables.

**Results:** Vomiting after oral feeding occurred in 18 (20.9%) patients with cancer and in 7 (20.0%) patients with diverticular disease. An NG tube was reinserted in 13 (15.1%) patients in the cancer group and in 3 (8.6%) patients in the diverticular disease group. In patients with cancer, the duration of operation was associated with GI-2 (*P* = 0.011), whereas in patients with diverticulitis, the duration of operation was associated with GI-1 (*P* = 0.001) and GI-2 (*P* = 0.044). In the diverticulitis group, a significant relationship was found between GI-2 and operative category (*P* = 0.03).

**Conclusion:** Longer operations led to more prolonged postoperative ileus after both laparoscopy and laparotomy, regardless of malignant or benign pathology. In anticipation of and/or following longer operations, surgeons should consider measures to shorten postoperative ileus.

## INTRODUCTION

Postoperative ileus (POI) is commonly characterized as transient impairment of bowel motility after abdominal surgery or other injury [1]. In POI, small bowel motility recovers between 24 and 48 hours after surgery, which is shorter than the 48–72 hours commonly required for recovery of colonic function [2, 3]. Clinically, POI presents as intolerance of oral feeding, distended abdomen, nausea and/or vomiting, diminished or absent bowel sounds and failure of passage of flatus and/or bowel movement [4]. Although bowel auscultation may reveal an end point of POI, bowel sounds may also result from small bowel activity rather than colonic function; thus, auscultation may lack reliability [1]. Flatus could also perhaps designate an end point of POI; however, questions have been raised about the relation of flatus and the end point of POI [3, 5]. The morbidity associated with POI delays oral feeding, prolongs hospitalization, and significantly increases risk of leak [6]. Ileus is the most common cause for delayed hospital discharge after abdominal surgery [7]. Different mechanisms have been hypothesized to explain the pathogenesis of POI, including neurogenic pathways [4]. Sympathetic overactivity seems to play an important role in this pathway. As reported in the literature, factors that induce this pathway are operative time and trauma [6–8]. Whereas colonic surgery is accompanied by longer POI periods, conflicting data exist regarding the effect of operative time on POI [2, 7–9]. In this cross-sectional study, we evaluated the effects of operative method and time on outcomes of patients with POI following surgeries for colorectal cancers and diverticulitis.

## METHODS AND MATERIALS

After institutional review board approval, we evaluated the records of all patients who underwent elective operations for either colorectal cancer or diverticular disease from July to December 2009. All operations including open resections, laparoscopic-assisted, total laparoscopic and hand-assisted laparoscopic procedures and conversions were included in the study. Emergent operations were excluded. Pain management was identical in all patients and included a patient-controlled analgesic (PCA) device to receive intravenous opiate analgesics. The PCA device delivered both continuous and demand doses for 1 day and then demand doses for an additional 1 or 2 days. Ultimately, patients received oral or injectable non-opioid analgesics for pain relief.

All patients had an orogastric or a nasogastric (NG) tube through the operation, which was removed at the termination of the procedure. Patients were allowed to drink clear liquids immediately after surgery and were monitored for the return of bowel function. The diet was advanced to a regular diet after bowel function returned and in the absence of abdominal distension and nausea or vomiting. The occurrence of two or more episodes of vomiting 200 cc or more per 24 hours in the absence of flatus or bowel movement warranted reinsertion of an NG tube. All patients were regularly monitored on a daily basis for return of bowel function, as well as nausea, vomiting, abdominal distension, pain medication use, and the need for NG tube reinsertion.

The following data were recorded from the patients’ charts: age, gender, total operative time, incision length, operative technique, previous abdominal surgery, ambulation activity and duration of hospitalization. The GI-1 end point was the postoperative time to toleration of clear liquids. The GI-2 end point was the postoperative time to the first bowel movement or flatus and toleration of a regular diet. Data were analysed using SPSS software (SPSS, Inc; Chicago, IL, USA).

## RESULTS

A total of 121 patients were included in the study; 62 (51.2%) were male and 59 (48.8%) were female. There was no significant difference in age between patients presenting with cancer (63.1 ± 13.7 years) and diverticulitis (53.7 ± 12.7 years). As shown in Table 1, 86 and 35 patients comprised the cancer and diverticular disease groups, respectively. Table 2 presents the numbers of patients who underwent different operative methods in each group. Laparoscopic procedures were performed on 41.9% and 22.9% of patients in the cancer and diverticular disease groups, respectively. Patients with diverticulitis were found to have intraperitoneal adhesions in 25.7% of cases, compared to 7.0% of patients with cancer (*P = *0.011). Intraoperative complications were noted in 1 (2.9%) patient in the diverticular disease group and 2 (2.4%) patients in the cancer group, including 1 enterotomy in each patient and 1 minor splenic injury in the latter group (*P = *0.584). The numbers of patients who underwent different operations are presented in Table 3.

**Table 1:** Patient demographics

	Cancer	Diverticulitis	*P*-value
**Gender** - *n* (%)			
M	48 (55.8)	14 (40.0)	*P*1 = 0.168
F	38 (44.2)	21 (60.0)	
**Age** (years)			
Mean ± S.D.	63.1 ± 13.7	53.7 ± 12.7	
Median	65.0	52.0	*P*2 < 0.001
Range	29–96	21–81	
Total of patients	86	35	
**Height (inches)**			
Mean ± S.D.	66.5 ± 4	66.3 ± 4.1	
Median	67.0	67.0	*P*2 = 0.773
Range	59–76	60–83	
Total of patients	86	35	
**Weight (pounds)**			
Mean ± S.D.	170.2 ± 41.2	176.6 ± 32.4	
Median	166.0	180.0	*P*2 = 0.408
Range	98–320	103–255	
Total of patients	86	35	
**Body Mass Index**			
Mean ± S.D.	26.9 ± 5.4	28.4 ± 5.1	
Median	26.0	28.1	*P*2 = 0.177
Range	16.8–43.3	18.8–40	
Total of patients	86	35	

p1: Chi-Squared Test

p2: Student's *t*-Test

**Table 2:** Operative details

	Cancer	Diverticulitis	*P*-value
**Operative Procedure** - *n* (%)			
Open	33 (38.4)	14 (40.0)	
Laparoscopic assisted	25 (29.1)	5 (14.3)	
Hand assisted	11 (12.8)	11 (31.4)	*P* = 0.124
Total laparoscopic	11 (12.8)	3 (8.6%)	
Laparoscopic converted	6 (7.0)	2 (5.7%)	
Total number of patients	86	35	

p: Likelihood ratio test

**Table 3:** Resection

	Cancer	Diverticulitis	*P*-value
**Resection** - *n* (%)			
Right	31 (36.0)	0 (0.0)	
Left	2 (2.3)	2 (5.7)	
Sigmoid	16 (18.6)	31 (88.6)	
Proctectomy	15 (17.4)	0 (0.0)	*P* < 0.001
Low anterior	8 (9.3)	1 (2.9)	
Abdominoperineal resection	13 (15.1)	0 (0.0)	
Transverse	1 (1.2)	0 (0.0)	
Total abdominal colectomy	0 (0.0)	1 (2.9)	
Total number of patients	86	35	

p: Likelihood ratio test

**Figure 1:**
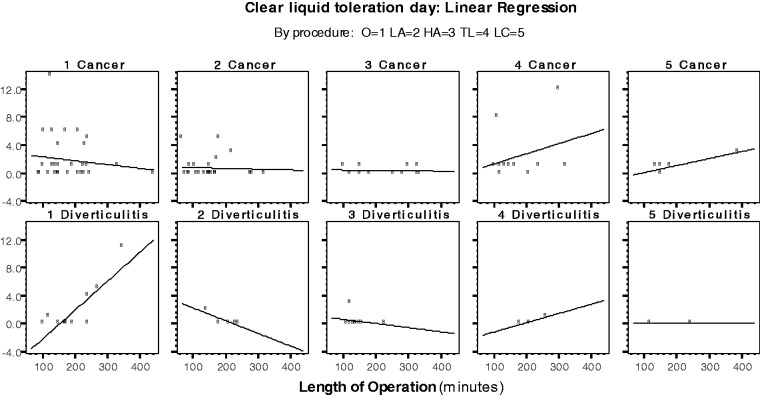
Linear regression analysis: clear liquid toleration day (GI-1) by population type and procedure.

**Figure 2:**
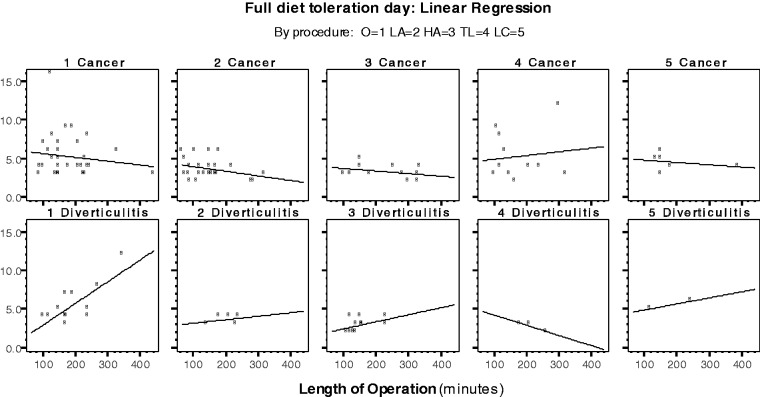
Linear regression: full diet tolerance day (GI-2) by population type and procedure.

**Figure 3:**
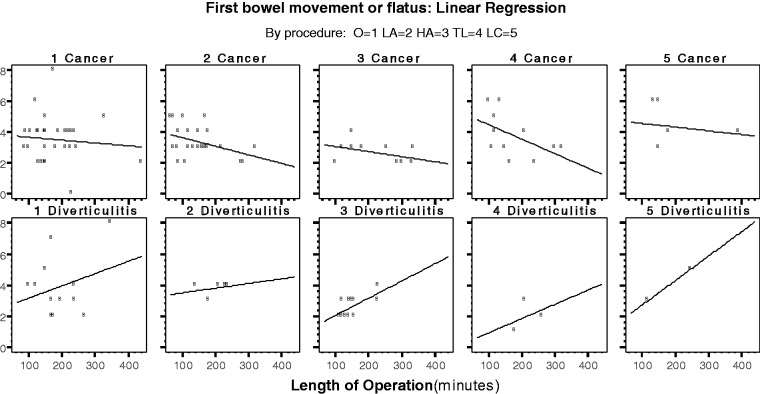
Linear regression: first bowel movement or flatus (GI-2) by population type or procedure.

The incision length was similar between the two groups (*P = *0.872). Postoperative pain was managed by PCA in 100% of patients with cancer and in 94.3% of patients with diverticulitis (*P = *0.082). All of the patients with diverticulitis ambulated early (the evening after surgery), while 87.2% of the patients with cancer were considered for early ambulation (*P = *0.019). The results are shown in Tables 4, 5 and 6 and Figures 1, 2 and 3.

**Table 4:** Linear regression: clear liquid toleration day by population type (GI-1)

Factor	Coefficient	CI (95%)	*P*-value
**Cancer: linear regression**			
Length of operation	0.000	−(0.007; 0.008)	0.907
Procedure	−0.078	−(0.514; 0.357)	0.721
R-Square = 0.002			
p-value (regression) = 0.935			
**Diverticulitis: Linear regression**			
Length of operation	0.020	(0.008; 0.032)	0.001
Procedure	−0.389	−(0.927; 0.148)	0.149
R-Square = 0.35			
p-value (regression) = 0.0016			

Dependent variable: Clear liquid toleration day

**Table 5:** Linear regression: full diet tolerance day by population type

Factor	Coefficient	CI (95%)	*P*-value
**Cancer: linear regression**			
Length of operation	−0.003	−(0.009; 0.003)	0.343
Procedure	−0.130	−(0.517; 0.257)	0.506
R-square = 0.018			
p-value (regression) = 0.475			
**Diverticulitis: Linear regression**			
Length of operation	0.019	(0.008; 0.029)	0.001
Procedure	−0.527	−(1.000; −0.053)	0.030
R-square = 0.415			
p-value (regression) = 0.0003			

Dependent Variable: Full diet toleration day

**Table 6:** Linear regression: first bowel movement or flatus by population type

Factor	Coefficient	CI (95%)	*P*-value
**Cancer: linear regression**			
Length of operation	−0.004	−(0.008; −0.001)	0.011
Procedure	0.107	−(0.098; 0.312)	0.301
R-square = 0.082			
p-value (regression) = 0.029			
**Diverticulitis: Linear regression**			
Length of operation	0.009	(0.000; 0.017)	0.044
Procedure	−0.321	−(0.709; 0.067)	0.101
R-square = 0.466			
P-value (regression) = 0.026			

Dependent variable: first bowel movement or flatus

In patients with cancer, the duration of the operation was associated with time to first bowel movement or flatus (GI-2; *P = *0.011). In the diverticulitis group, the duration of the operation was associated with the ability to tolerate clear liquid (*P = *0.001), time to first bowel movement or flatus (GI-2; *P = *0.044) and time to full diet toleration (GI-2; *P = *0.001). Data were also analysed to evaluate whether the type of surgical procedure was associated with POI outcomes. Although no associations were found in patients with cancer, a significant relationship was found in patients with diverticular disease between regular diet toleration (GI-2) and procedure (*P = *0.03). The incidence of vomiting after oral feeding was similar between groups, 20.9% and 20.0%, respectively; however, the rate of NG tube reinsertion was higher in the cancer group (15.1% vs 8.6%).

## DISCUSSION

Many surgeons have traditionally believed that the duration of POI is proportional to the magnitude and duration of surgery performed [4, 6], although this view has been questioned by some authors [2, 9]. Longer operations may reflect more complicated surgeries and extensive resections.

The classical management of POI has primarily been by routine NG tube decompression of the GI tract [10]. This method was recently supplemented by a selective approach [10]. Further studies demonstrated the ineffectiveness of NG tube decompression in reducing POI [6, 11]. It became clear that early feeding during the first two days after surgery could have advantageous effects and shorten the duration of POI [6]. Although this practice was initially introduced for laparoscopic operations, Binderow *et al.* demonstrated its feasibility for open colorectal resections [10].

Early oral feeding is now considered a standard of care in many centers [6, 7, 10]. However, laparoscopic colectomy shortens length of stay and POI [6, 12]. Postoperative use of morphine adversely affects GI motility; however, Cali *et al.* did not find any relation between incision length and bowel function [13]. Although any salutary effect of ambulation on POI may be more perceived than real [14], early ambulation is recommended because of its beneficial effects on the pulmonary and the vascular system and an association with decreased morbidity [6]. The positive effect of preoperative suggestion in a believable manner was studied by Disbrow *et al.* and seems to have a beneficial effect on POI [15].

The concept of multimodality rehabilitation or fast-track surgery as an important approach to shortening the postoperative recovery and hospital stay has been received enthusiastically [6, 16–18]. These protocols have been shown to reduce the duration of POI to 48 hours and the hospital stay to 2–4 days [16].

It is a common practice to use opioid analgesics after any surgery, leading to longer POI periods. Therefore, peripherally acting µ-opioid receptor antagonists, such as the oral drug alvimopan, have been used to reduce POI [6, 19, 20]. The effect of alvimopan has been well studied [6, 19–21].

The current retrospective study was undertaken in order to evaluate the effect of operative time and method on POI. Those patients who underwent elective colorectal resections—either by open, laparoscopic or hand-assisted techniques—were included. Emergent operations were excluded due to the unfavorable impact of emergent disease on postoperative GI motility. Based on the disease category, the patients were placed in one of two groups: cancer or diverticulitis. The higher rate of laparoscopic procedures performed in the cancer group as compared to the diverticular disease group may be due to a higher rate of peritoneal adhesions found in the latter group. Thus, not surprisingly, the operative times were longer in the diverticular disease group. The current study indicated a significant relationship between operative times and both the GI-1 and GI-2 end points in the diverticular disease group and the GI-2 end point in the cancer group.

## CONCLUSION

In agreement with findings from previous studies, we suggest that operative time correlates with prolonged POI in patients presenting with either diverticulitis or cancer. Results of our study reinforce the idea that the type of procedure has a relationship with POI in patients with diverticulitis; however, the same relationship was not found in the cancer group. Further investigations are needed to identify more effective ways to reduce the duration of POI.

## ACKNOWLEDGMENTS

The authors thank Paula Strassmann and Maristela Percivale for their assistance with the study design and statistical evaluation.

**Conflict of interest:** none declared.

## References

[1] Holte K, Kehlet H (2000). Postoperative ileus: a preventable event. Br J Surg.

[2] Livingston EH, Passaro EP (1990). Postoperative ileus. Dig Dis Sci.

[3] Luckey A, Livingston E, Tache Y (2003). Mechanisms and treatment of postoperative ileus. Arch Surg.

[4] Schwenk W, Bohm B, Haase O (1998). Laparoscopic versus conventional colorectal resection: a prospective randomised study of postoperative ileus and early postoperative feeding. Langenbecks Arch Surg.

[5] Waldhausen JH, Shaffrey ME, Skenderis BS (1990). Gastrointestinal myoelectric and clinical patterns of recovery after laparotomy. Ann Surg.

[6] Kehlet H (2008). Postoperative ileus: an update on preventive techniques. Nat Clin Pract Gastroenterol Hepatol.

[7] Baig MK, Wexner SD (2004). Postoperative ileus: a review. Dis Colon Rectum.

[8] Bauer AJ, Boeckxstaens GE (2004). Mechanisms of postoperative ileus. Neurogastroenterol Motil.

[9] Condon RE, Frantzides CT, Cowles VE (1986). Resolution of postoperative ileus in humans. Ann Surg.

[10] Binderow SR, Cohen SM, Wexner SD (1994). Must early postoperative oral intake be limited to laparoscopy?. Dis Colon Rectum.

[11] Wolff BG, Pembeton JH, van Heerden JA (1989). Elective colon and rectal surgery without nasogastric decompression. A prospective, randomized trial. Ann Surg.

[12] Chen HH, Wexner SD, Iroatulam AJ (2000). Laparoscopic colectomy compares favorably with colectomy by laparotomy for reduction of postoperative ileus. Dis Colon Rectum.

[13] Cali RL, Meade PG, Swanson MS (2000). Effect of morphine and incision length on bowel function after colectomy. Dis Colon Rectum.

[14] Waldhausen JH, Schirmer BD (1990). The effect of ambulation on recovery from postoperative ileus. Ann Surg.

[15] Disbrow EA, Bennett HL, Owings JT (1993). Effect of preoperative suggestion on postoperative gastrointestinal motility. West J Med.

[16] Kehlet H (2008). Fast-track colorectal surgery. Lancet.

[17] Kehlet H, Dahl JB (2003). Anaesthesia, surgery, and challenges in postoperative recovery. Lancet.

[18] Kehlet H, Wilmore DW (2008). Evidence-based surgical care and the evolution of fast-track surgery. Ann Surg.

[19] Delaney CP, Wolff BG, Viscusi ER (2007). Alvimopan, for postoperative ileus following bowel resection: a pooled analysis of phase III studies. Ann Surg.

[20] Wolff BG, Weese JL, Ludwig KA (2007). Postoperative ileus-related morbidity profile in patients treated with alvimopan after bowel resection. J Am Coll Surg.

[21] Tan EK, Cornish J, Darzi AW (2007). Meta-analysis: Alvimopan vs placebo in the treatment of post-operative ileus. Aliment Pharmacol Ther.

